# A mechano-chemiosmotic model for the coupling of electron and proton transfer to ATP synthesis in energy-transforming membranes: a personal perspective

**DOI:** 10.1007/s11120-014-0043-3

**Published:** 2014-09-30

**Authors:** Eldar A. Kasumov, Ruslan E. Kasumov, Irina V. Kasumova

**Affiliations:** Research and Production Centre «KORVET», Moscow Region, Domodedovo Russia

**Keywords:** F_0_F_1_-ATPase, *γ*-Subunit rotation, Mechano-chemiosmotic model, Shrinkage–swelling, Electron transfer

## Abstract

ATP is synthesized using ATP synthase by utilizing energy either from the oxidation of organic compounds, or from light, via redox reactions (oxidative- or photo phosphorylation), in energy-transforming membranes of mitochondria, chloroplasts, and bacteria. ATP synthase undergoes several changes during its functioning. The generally accepted model for ATP synthesis is the well-known rotatory model (see e.g., Junge et al., Nature 459:364–370, 2009; Junge and Müller, Science 333:704–705, 2011). Here, we present an alternative modified model for the coupling of electron and proton transfer to ATP synthesis, which was initially developed by Albert Lester Lehninger (1917–1986). Details of the molecular mechanism of ATP synthesis are described here that involves cyclic low-amplitude shrinkage and swelling of mitochondria. A comparison of the well-known current model and the mechano-chemiosmotic model is also presented. Based on structural, and other data, we suggest that ATP synthase is a Ca^2+^/H^+^–K^+^ Cl^−^–pump–pore–enzyme complex, in which *γ*-subunit rotates 360° in steps of 30°, and 90° due to the binding of phosphate ions to positively charged amino acid residues in the N-terminal *γ*-subunit, while in the electric field. The coiled coil *b*_2_-subunits are suggested to act as ropes that are shortened by binding of phosphate ions to positively charged lysines or arginines; this process is suggested to pull the *α*_3_ *β*_3_-hexamer to the membrane during the energization process. ATP is then synthesized during the reverse rotation of the *γ*-subunit by destabilizing the phosphated N-terminal *γ*-subunit and *b*_2_-subunits under the influence of Ca^2+^ ions, which are pumped over from storage—intermembrane space into the matrix, during swelling of intermembrane space. In the process of ATP synthesis, energy is first, predominantly, used in the delivery of phosphate ions and protons to the *α*_3_ *β*_3_-hexamer against the energy barrier with the help of C-terminal alpha-helix of *γ*-subunit that acts as a lift; then, in the formation of phosphoryl group; and lastly, in the release of ATP molecules from the active center of the enzyme and the loading of ADP. We are aware that our model is not an accepted model for ATP synthesis, but it is presented here for further examination and test.

## Introduction

**Fig. 1:**
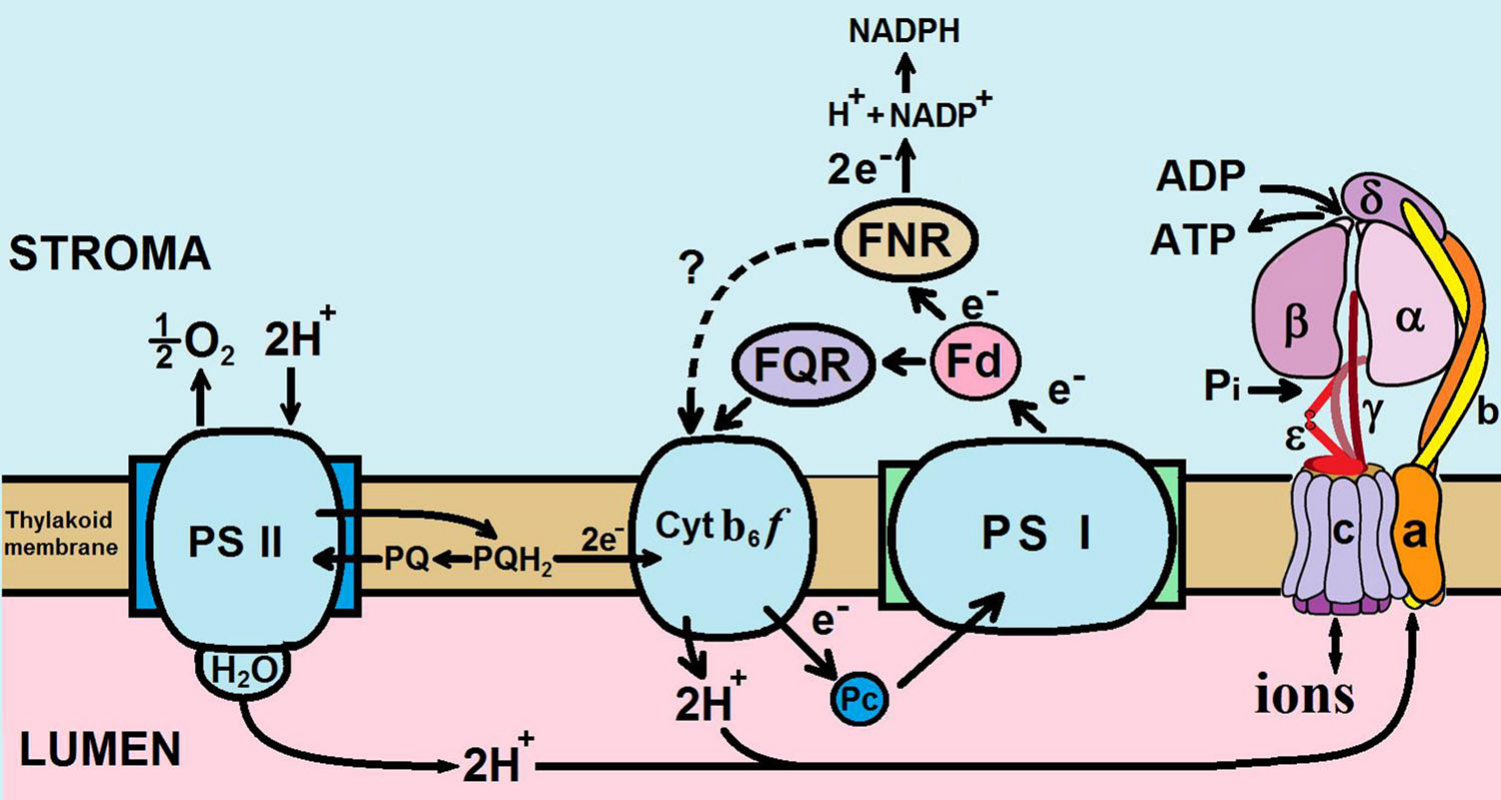
A scheme of electron transport chain in chloroplasts and arrangement of protein complexes (Photosystem I, Photosystem II, cytochrome *b*_6_ *f*, *FQR* ferredoxin (plasto)quinone-reductase, *Fd* ferredoxin, *FNR* ferredoxin-NADP-oxidoreductase and ATP synthase subunits in *E. coli*) in the thylakoid membrane. This figure was modified by the authors from the drawings by Tikhonov (2013a, b); and http://www.atpsynthase.info/Gallery.html

Energy transformation is a fundamental process needed for existence and activity of living organisms. ATP is a major chemical energy-containing intermediate, which release energy upon hydrolysis; the other intermediate is the reduced NADP, i.e., NADPH (Cramer and Knaff 1991). Most of ATPs are synthesized by ATP synthases (F_0_F_1_-ATPases) in energy-transforming membranes of mitochondria, chloroplasts, and bacteria (see Junge 2013). In redox reactions, during respiration and photosynthesis, a vectorial transfer of charges occurs from the (electron) donor side to the (electron) acceptor side, along an electron transport chain (ETC) (see below and Fig. 1).

The steps in the inner membrane of mitochondria include electron transport from one intermediate to the next: dehydrogenase (Deh) → flavoprotein (Fp) → ubiquinone (CoQ) → cytochrome (Cyt) *bc*_1_→ Cyt *c*→ Cyt *aa*_3_. However, in the thylakoid membranes, electron transport begins at PSII, ending at NADP: photosystem II (PSII) → plastoquinone (PQ) → Cyt *b*_6_ *f*→ plastocyanin (PC) → photosystem I (PSI) → ferredoxin (Fd) → ferredoxin-NADP reductase (FNR). In both photosynthetic and respiratory electron transport pathways in the thylakoid membrane of cyanobacteria, they are Deh and PSII → PQ → Cyt *b*_6_ *f*→ PC → oxidase (Ox) and (PSI → Fd → FNR) (see Vermaas 2001). We note that Cyt *b*_6_ *f* or Cyt *bc*_1_ has a central position in the electron transport chain of all energy-transducing organelles and organisms, and it performs an important function.

Almost 90 years ago, Keilin (1925) showed that electrons derived from the activation of hydrogen atoms by dehydrogenases are transferred via three hemoproteins, which he named cytochrome (cyt) *a*, *cyt b*, and cyt *c*, to an oxygen-activating oxidase. Transfer of electrons through cytochromes up to oxygen is known to be accompanied by the formation of ATP from ADP and inorganic phosphate, as a result of oxidative phosphorylation (Cramer and Knaff 1991). Mechanism of phosphorylation during respiration has consistently attracted the attention of many researchers. Throughout the years, there have been different hypotheses, which have provided explanations for the transformation of liberated energy during substrate oxidation or during “photolysis” of water, in Photosystem II, into the energy of chemical bonds in a molecule of ATP.

Lipmann (1946) was the first to propose a chemical coupling hypothesis for ATP synthesis, where the reduced electron carrier promotes the formation of active phosphoryl group by nucleophilic attack, and this phosphoryl group is then associated with ADP. Later, Slater (1953) modified this chemical hypothesis, where the phosphate ion is not directly connected to the carrier of the electron transport chain and the energy conversion is carried out in a series of sequential reactions involving high-energy intermediates, which is, in general, similar to substrate phosphorylation. However, despite extensive pursuit, which has lasted for many years, phosphorylated intermediates could not be found.

Boyer (1964) presented another hypothesis for ATP synthesis; it was a conformational change hypothesis, which assumed that energy storage takes place by conformational changes in proteins of the electron transport chain, involving “contraction and relaxation” of the enzyme, similar to the well-known actin–myosin system. In some ways, this conformational hypothesis is reminiscent of the chemical hypothesis; it had its adherents (Lehninger 1966; Harris et al. 1968), who were attracted by the existence of mitochondrial conformational changes during oxidative phosphorylation. According to one view of the conformational hypothesis, ATP, per se, does not have energy to give, and energy is not needed for ATP synthesis in vivo (Banks and Vernon 1970). In agreement with this version of conformational hypothesis, Paul Boyer later suggested that energy is not required for ATP synthesis, and energy of protein conformational changes, coupled with the membrane potential and protonation–deprotonation, is actually used for the release of ATP from ATPase/ATP synthase (Boyer 1975).

The generally accepted view is that of the universally accepted chemiosmotic hypothesis, proposed by Mitchell (1961). According to this hypothesis, energy for ATP synthesis ultimately comes from the proton-motive force (pmf), which includes both a pH gradient and membrane potential. The pmf is calculated as follows: During both respiration and photosynthesis, vectorial electron transfer occurs from (electron)donors to (electron)acceptors occurs across the mitochondrial and thylakoid membranes. This results in separation of positive and negative charges on the opposite sides of the membranes, where protons (H^+^) are accumulated in the intermembrane space of mitochondria or in thylakoids and hydroxyl (OH^−^) in the matrix or stroma, respectively. Further, Mitchell (1961) suggested that ATP is synthesized by ATP synthase (H^+^-F_0_F_1_-ATP synthase), by using energy in the form of ΔμH^+^ (Δψ−—electric potential and ΔpH—proton gradient). Asymmetric organization of the electron transport chain in the form of three loops contributes to the proton-motive force (Mitchell 1966; cf. Spetzler et al. 2012).

Later, this chemiosmotic hypothesis was refined in terms of proton transfer (Mitchell 1974), and the idea was proposed that the hydrophobic component of ATPase-F_0_ is oligomycin sensitive proton channel through the membrane. Thus, F_1_ is attached to F_0_ so that its active center faces the proton channel F_0_. As a result of ATP hydrolysis, the protons are released into the channel, while ADP and Pi are transported back through F_1_ to the aqueous phase. However, in the synthesis of ATP, protons are picked up from the proton channel by phosphate, and phosphorylation of ADP occurs in the active center of F_1_.

In our view, the unchallenged acceptance of chemiosmotic hypothesis has led to the fact that rational parts of chemical and conformational hypothesis have been rejected completely. The modified conformational hypothesis of Boyer (1975) was, however, a part of the chemiosmotic hypothesis, since conformational changes of the enzyme were occurring as a result of proton transfer, and then conformational hypothesis itself, in this form, has become a dogma. The mechanism that underlies this fundamental process still remains obscure.

We quote Walker (2013):

Overall architecture, organization and mechanistic principles of the ATP synthases are mostly well established, but other features are less well understood. For example, ATP synthases from bacteria, mitochondria, and chloroplasts differ in the mechanisms of regulation of their activity, and the molecular bases of these different mechanisms and their physiological roles are only just beginning to emerge. Another crucial feature lacking a molecular description is how rotation driven by Δp is generated, and how rotation transmits energy into the catalytic sites of the enzyme to produce the stepping action during rotation. One surprising and incompletely explained deduction based on the symmetries of c-rings in the rotor of the enzyme is that the amount of energy required by the ATP synthase to make an ATP molecule does not have a universal value

In this regard, a remark of Wolfgang Junge is also very relevant: “Is our knowledge on the ion-driven and rotary ATP synthase now ready and finished? Not at all, because a full structure of F_0_F_1_ at atomic resolution is not yet available, and the structural and dynamic knowledge has been assembled from different sources” (Junge 2013).

## Structure of ATP synthase

ATP synthase catalyzes phosphorylation of ADP by inorganic phosphate using energy available from electrochemical potential ΔµH^+^; further, under certain conditions, the same enzyme can hydrolyze ATP, for example, during high-amplitude swelling of mitochondria (Lehninger 1959). ATP synthase is a large complex in mitochondria, in chloroplasts, and in respiring and photosynthesizing bacteria; in *E. coli*, it has a molecular mass of 593–665 kDa. Structures of bacterial and chloroplast enzymes are almost identical, although the mitochondrial complex contains several additional subunits (Kagawa et al. 2000; Pedersen et al. 2000; Varco-Merth et al. 2008).

**Fig. 2:**
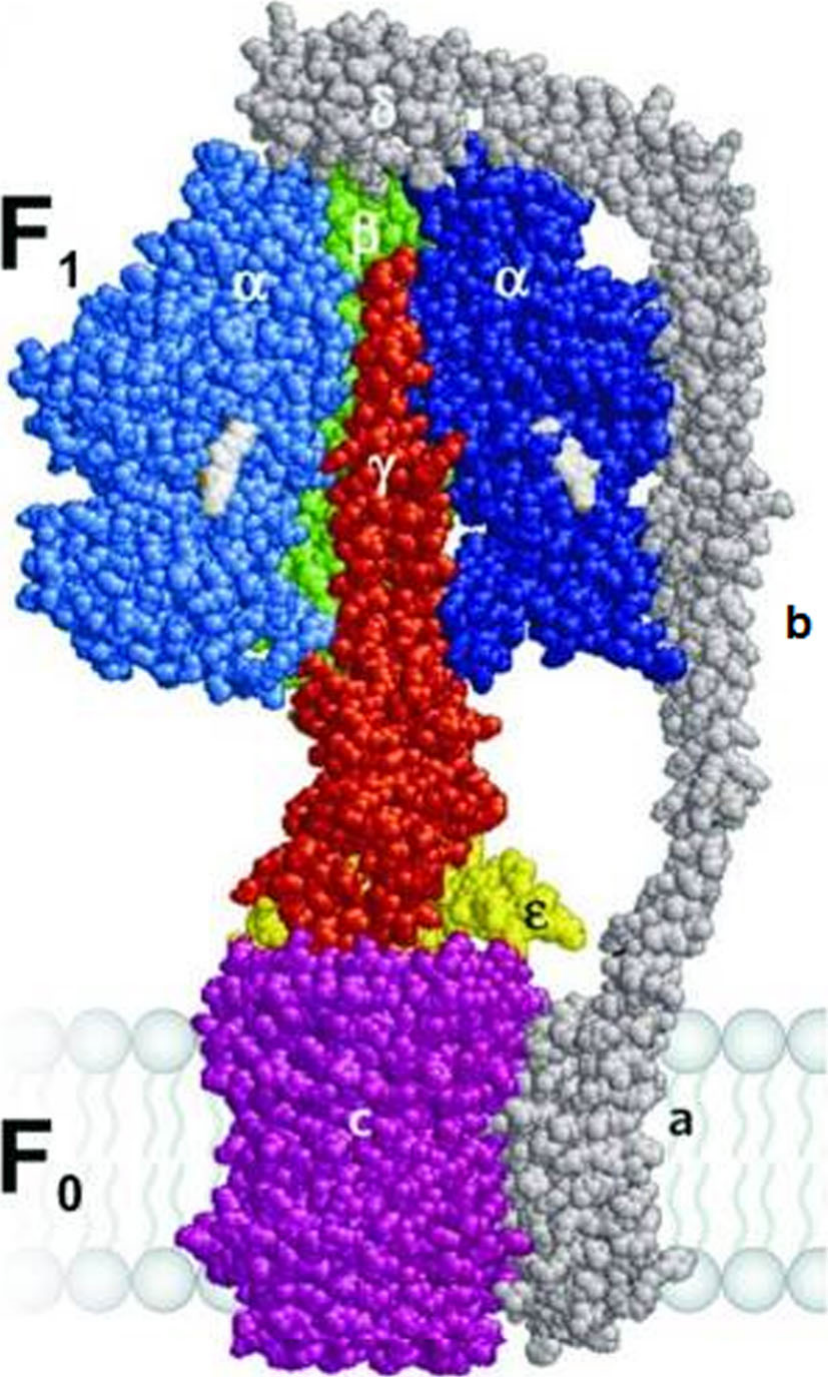
Structure of ATP synthase in membrane. Symbols—*α*, *β*, *γ*, *δ*, *ε*, *b*_2_, *a*, indicate subunits of ATP synthase. Space-filling homology model of EF_0_F_1_ with one *α*-subunit and two *β*-subunits removed to expose the γ-subunit (*red*) in the center of the (*αβ*)3-pseudohexagon, with *α* shown in *blue*, *β* in *green*, *γ* in *red*, *ε* in *yellow*, *δ* (*on top*) and subunits *a* and *b* in *dark gray*, nucleotides in *pale gray*, and *c*10 in *magenta*. The bulge of subunit *γ* (made up of the convex coiled coil consisting of the N- and C-terminal helices of *γ*) is not visible because it points away from the viewer. This figure is reproduced from Wächter et al. (2011) with the permission of Wolfgang Junge

Structure of *E. coli* ATP synthase is shown schematically in Fig. 2. The ATP synthase with a *head* of 9 nm in diameter is located on the membrane, and it protrudes ~3 nm toward the matrix, stroma, or cytoplasm (Racker 1979; Skulachev 1989; Greie et al. 2000). The head of ATP synthase in *E. coli*, F_1_, consists of *α*_3_ *β*_3_ *γδε*-subunits (Kagawa et al. 2000; Varco-Merth et al. 2008). Three regulatory nucleotide-binding *α*-subunits (Malyan 2007, 2013) and three catalytic *β*-subunits form a symmetrical hexamer, while the *δ*-subunit is in the form of a *lid*-*cap* on the apical part of this hexamer. The *b*-subunit consists of 156 residues and forms an elongated dimer extending from the periplasmic side of the cytoplasmic membrane to the top of F_1_, where it interacts with a *δ*-subunit. The transmembrane domain is formed by residues 1–24, the dimerization domain includes residues 53–122, and the C-terminal *δ*-binding domain is made of residues 123–156. The interaction of *b*_2_ with F_1_ occurs predominantly through the C-terminal *δ*-binding domain. Although interactions exist between *b* and *α*_3_ *β*_3_-hexamer, *δ*-subunit is required for the binding of F_1_ to F_0_ in membranes. The interaction of *b*_2_- and, *δ*-subunits, mediated through the C-terminal regions of each subunit, appears to be the key to this binding. The tether region (residues 25–52) links the transmembrane and dimerization domains; its function is not well understood, but it is known to interact with the *alpha* subunit and to play a role in coupling of proton translocation to catalysis. It was revealed that the dimerization domain of *b* contains a two-stranded right-handed coiled coil with offset helices (Wood and Dunn 2007, also see references therein).

Asymmetrical *γε*-subunit complex links the hexamer with the transmembrane part – F_0_. The *ε*-subunit is known to inhibit the hydrolysis of ATP. This inhibitory effect is modulated by conformation of C-terminal alpha-helices of epsilon (Vik 2000), and the “extended” but not “hairpin-folded” state is responsible for the inhibition of ATP synthase (Suzuki et al. 2003, Iino et al. 2009). Two long N- and C-terminal helices of *γ*-subunit form a twisted helical element that extends into the *α*_3_ *β*_3_-hexamer, acting as a rotating spindle imparting structural asymmetry of the catalytic sites (Abrahams et al. 1994; Samra et al. 2006). F_0_ is composed of 8–15 *c*-subunits and asymmetrical *ab*_2_-subunits, where the exact number of *c*-subunits depends on the organism. In chloroplasts, Subunits I and II correspond to subunit *b* in *E. coli*, whereas subunits III and IV of chloroplasts correspond to subunits *c* and *a* of *E. coli* (Varco-Merth et al. 2008). Mitochondrial F_0_ contains additional subunits: *d*, *e*, *f*, *g*, (F6) 2, A6L, and OSCP (oligomycin sensitivity-conferring protein) (Pedersen et al. 2000) unlike that of *E. coli*.

Hairpins are formed by two transmembrane helices and loops with conserved amino acid residues on the matrix side. Monomers of *c*-subunits form the double ring (inner—F_0_c_1_ and outer—F_0_c_2_) in the membrane, and the number of monomers varies depending on the source (Varco-Merth et al. 2008).

## Rotation of the γ-subunit

According to the currently accepted model, the *γ*-subunit rotates inside the *α*_3_ *β*_3_-hexamer, changing conformation of *β*-subunits, which leads to the formation of different states in relation to nucleotide binding (Boyer 1997; Walker 2013). Rotation of *γ*-subunit in counterclockwise direction in ATP hydrolysis (Noji et al. 1997; Sielaff et al. 2008; Kagawa et al. 2000; Sambongi et al. 2000) has been established by the use of biochemical, and spectroscopic methods. Evron et al. (2000), Vik (2000) and Sambongi et al. (2000) have suggested that the *γ*-subunit acts in cooperation with the *ε*-subunit; it is also known that the *γ*-subunit undergoes conformational changes in the presence of ATP and applied transmembrane potentials (Samra et al. 2006). In our view, although the fact of rotation of subunits during ATP synthesis has not been confirmed experimentally, yet it has been suggested that *γ*-subunit will rotate in clockwise direction during ATP synthesis (Sambongi et al. 2000). Evidence for the chemical synthesis of ATP, driven by mechanical energy, was shown by Itoh et al. (2004) when they attached a magnetic bead to the *γ*-subunit of isolated F_1_ on a glass surface, and had rotated the bead using electrical magnets. The clockwise rotation of *γ*-subunit (with the F_1_-ATPase molecule viewed from the side of the *γ*-subunit projecting from *α*_3_ *β*_3_) promoted ATP synthesis from ADP and P_i._ Rotation of the *γ*-subunit in the opposite direction was associated with ATP hydrolysis.

The mechanism of *γ*-subunit rotation resembles the rotation of a thread composed of twisted carbon nanotubes in electric field when electrolytes are present (Foroughi et al. 2011); however, unlike carbon nanotubes, *γ*-subunit is surrounded by other proteins, with different structures. Those authors who have constructed a nanomotor have suggested that a hydrostatic motor mechanism may provide an explanation for simultaneous longitudinal shrinkage and torsion rotation during the increase of the volume of the thread, mentioned above (Foroughi et al. 2011). Furthermore, hydrostatic (or quasi-hydrostatic) pressure is created by the changes in relative concentration of ions of opposite charges in the thread volume in order to compensate for the electric charge applied to nanotubes. Therefore, it is possible to make the following conclusions regarding the principles of this motor functioning on the basis of the work of Foroughi et al. (2011): (1) helical angles of the thread relative to the axis should be less than 54.73°; (2) rotation will not proceed, if helices have opposite chirality; hence, helices must possess the same chirality in order to rotate the thread; (3) maximum rotation angle depends on the thread length; (4) by switching between modest inter-electrode potentials (0 V and −3 V at 1 Hz), a reversible paddle rotation of up to 180°, with a 65-nm length of actuating yarn 15-µm in diameter, is achieved; (5) lengthwise contraction and torsional rotation, during the yarn volume increase, is caused by electrochemical double-layer charge injection—(tetrabutylammonium hexafluorophosphate (TBA.PF6) in acetonitrile); and (6) reversibility of rotation depends on the place of thread fixation.

According to the data of Kasumov et al. (1990), compression of a protein molecule takes place under low concentration of salts and this effect is associated with the action of anions (Volynskaya et al. 2006, 2007). It is known from the work of von Hippel and Schleich (1973) and Han et al. (2011) that the stabilizing effect of anions is decreased as in the Hofmeister series: citrate > sulfate > phosphate > chloride > nitrate > thiocyanate. Monovalent cations are maximally effective in this case and, on the contrary, polyvalent cations interfere with the action of polyvalent anions. Further, calcium and manganese ions exhibit destabilizing action (von Hippel and Schleich 1973).

Structure of chirally twisted C-terminal and N-terminal alpha-helices (Feniouk and Junge 2005; Samra et al. 2006) in *γ*-subunit resembles a thread made of carbon nanotubes and the helical angle of N-terminal helix in relation to C-terminal helix is about 40°. The globular part of the *γ*-subunit, together with *ε*-subunit, makes a contact with the conserved loop of the *c*-ring, thereby closing a part of the *c*-ring entry.

Free phosphate plays an important role in counteracting inhibition by ADP. Slow deactivation of ATP hydrolysis occurs after pmf dissipation; it is accelerated by ADP and slowed down by phosphate, indicating that the enzyme shifts back to the “ADP-inhibited” state. Reactivation of enzyme can be achieved by forced rotation of *γ*-subunit from the “ADP-inhibited” position (Feniouk and Junge 2005).

ATP (when dealing with ATP hydrolysis) or electrochemical potential—ΔµH^+^ (when dealing with ATP synthesis) is the source of energy for rotation of the *γ*-subunit. Okazaki and Hummer (2013) used molecular dynamics simulation to construct the first conformation of the intermediate state following the 40° sub-step of rotary motion, and to study the timing and molecular mechanism of inorganic phosphate (Pi) release coupled to rotation. We note that during hydrolysis, ATP is the source of ADP, Pi, and protons. ADP and Pi as polyvalent anions are the stabilizing agents of globular proteins, including the *γ*-subunit. Consequently, on the one hand, ADP and Pi are the reaction products of enzymatic catalysis, but on the other hand, they are involved in rotation of the *γ*-subunit.

In our opinion, the above discussion on the dependence of rotation on the place of fixation leads us to doubt the correctness of the interpretation of experiments on the *γ*-subunit and *c*-ring joint rotation, because, as we assume, these rotations cannot proceed under natural conditions with membrane-bound ATPase.

## Rotation of F_0_c-ring

It is known that F_0_c-ring rotates differently under different conditions. Pänke et al. (2000) observed counterclockwise rotating actin filaments, attached to the C-terminal end of subunit *c* in the presence of MgATP, when EF_0_-EF_1_ was immobilized on a Ni-NTA-coated glass slide by the engineered His-tag at the N-terminus of subunit beta. We believe that experiments with actin filaments do not provide firm conclusions since the actin filaments are polymerized in chains of monomers, which themselves undergo a range of chemical events such as ATP hydrolysis, polymerization, and depolymerization (Yogurtcu et al. 2012). We note that phosphate, MgATP, and MgADP act on the tension and stiffness of the fiber (Lu et al. 2005). The mechanical behavior depends on the chemical state of actin. ADP-actin filaments are softer than ATP actin (Carlsson 2010).

In experiments of Ishmukhametov et al. (2010), biotinylated n-F_0_F_1_ was attached to a cover slip via 6× His-tags on the *β*-subunit N-terminus. Subsequent addition of avidin-coated gold nanorods then became bound to the biotins positioned on the *c*-ring distal from F_1_. Nanorods observed were specifically bound to the *c*-ring of F_0_F_1_ on the microscope slide as n-F_0_F_1_ (Ishmukhametov et al. 2010). The *c*-ring rotation occurs in steps of 36°. According to the principles of nanomotor of carbon nanotubes, rotation depends on the place of the attachment (Foroughi et al. 2011), e.g., if *α*_3_ *β*_3_-hexamer is attached, then the *γ*-subunit and *c*-ring can be rotated together without the membrane. Moreover, in these experiments, rotation is dependent on the concentration of PEG-400. If the *c*-ring is attached in membrane or artificially to a cover slip, then *γ*-subunit will rotate with the C-terminus at the top of the *α*_3_ *β*_3_-hexamer cavity as in a joint.

Wächter et al. (2011) presented experiments on the immobilization of EF_0_-EF_1_ and the attachment of a superparamagnetic bead to the stator. If the magnetic bead was attached to the N-terminal end of both copies of *b*, a rotating magnetic field twisted *b*_2_ around its long axis. Alternatively, if the bead was attached to the *c*_10_ ring properly, and if the *a*-subunit was locked to the *c*_10_ ring by a disulfide bridge, rotation around the long axis of the *c*_10_ ring flexed *b*_2_ rather than twisting it.

Tsunoda et al. (2001) noted that the rotation of the *c*-subunit is probably due to release of the *c*-subunit ring from its critical interaction with the *a*-subunit. Further, according Sambongi et al. (1999), rotation of the *c*-ring was lost rapidly. This can be explained if the *c*-subunit ring, once displaced from the *a*-subunit, is only weakly bound to the γɛ-subunits and is quickly released by the viscous drag due to the torque of the rotation (Tsunoda et al. 2000). It was shown that “the c subunit rotation could be observed in the presence of Triton X-100 but not in the absence of the detergent” (Sambongi et al. 1999; Tsunoda et al. 2000).

To investigate the sequential stepping of the proton-driven *c*-ring rotation, Duser et al. (2009) fused enhanced green fluorescent protein (EGFP) as fluorescence resonance energy transfer (FRET) donor to the C-terminus of the *a*-subunit. Alexa568-maleimide was used as a FRET acceptor and was covalently bound to a cysteine (E2C mutation) of one *c*-subunit. ATP synthase molecules were reconstituted singly into liposomes, which then had ATP synthesis activity. The radius of the FRET acceptor rotation was 2.5 nm on the *c*-ring. We suggest that the authors interpreted incorrectly a correct experiment that was performed in a membrane. Since, the FRET acceptor was covalently bound only to a cysteine (E2C mutation) of one *c*-subunit, then a FRET acceptor will bind at the first ATP synthase to the first *c*-monomer beginning from the *a*-subunit in a clockwise direction; at the second ATP synthase, it will bind to the second *c*-monomer, and at the third ATP synthase it will bind to the third *c*-monomer. At the same time, it is known that conformational changes of the subunit *c* occur during the protonation–deprotonation of Asp 61**(**Rastogi and Girvin 1999), and the structure of *c*-ring is deformed during energization of the membrane. In this case, the FRET signals will arrive at an average from all *c*-monomers of all ATP synthases as the integral signal, and a deformation of the structure of *c*-ring during energization was interpreted by the authors (Duser et al. 2009) as the *c*-ring rotation.

Thus, we suggest that all researchers must take into account, in their studies, for manipulation with the rotation of ATP synthase subunits, an absolute dependence of results on the place of attachment of the subunits. This conclusion is supported by experiments of Tanabe et al. (2001), where the *γε*c_10–14_-complex is a mechanical unit of the enzyme, and, it can be used as a rotor or a stator experimentally, depending on which subunit is immobilized. The membrane parts of F_0_c-subunits must be integrated exclusively in the membrane where they attach themselves naturally. Under these conditions, the *c*-subunit of the F_0_ part of ATP synthase has the ability for free conformational changes, where a conformational change of the *c*-ring might transform it to a non-selective pore (Chinopoulos and Szabadkai 2013). The *c*-subunit of the mitochondrial F_1_-F_0_ATP synthase, which is located on the all surfaces of inner membrane, has been recently found to be a fundamental component of the mitochondrial permeability transition pore—mPTP (Bonora et al. 2013; Alavian et al. 2014). The mPTP is a high-conductance channel that is located at the contact sites between the inner and outer mitochondrial membranes. The molecular composition of the mPTP is not yet clear, but several proteins have been shown to be its component that participate in mPTP activity, including voltage-dependent anion channels (VDAC), adenine nucleotide translocase (ANT), and the inorganic phosphate carrier (P_i_C) (De Marchi et al. 2014, and references therein). Ca^2+^ ions, prooxidant, and proapoptotic proteins, a decrease in the mitochondrial membrane potential, pH variations, and adenine nucleotides all sensitize the opening of the pore (Baumgartner et al. 2009). The mPTP channel is responsible for the non-selective permeability state of the mitochondrial inner membrane. De Marchi et al. (2014) observed that forcing mPTP opening or closing did not impair mitochondrial Ca^2+^ efflux. Therefore, in the opinion of the authors, their results strongly suggest that the mPTP does not participate in mitochondrial Ca^2+^ homeostasis in a physiological context in HeLa cells (De Marchi et al. 2014).

It is possible that calcium channel is formed by dimers of ATP synthase. Purified F-ATPsynthase dimers of yeast mitochondria display Ca^2+^-dependent channel activity with properties resembling those of the permeability transition pore (PTP) of mammals (Carraro et al. 2014). These results suggest that the yeast PTP originates from F-ATP synthase, and indicates that dimerization may be required for pore formation in situ.

## Current model of action mechanism of ATP synthase

The current consensus is, with supporting data, that ATP synthase consists of two motors (mechano-chemical motor—F_1_, working at the expense of energy released during ATP hydrolysis, and H^+^—managed electric motor—F_0_ that utilizes energy of the electrochemical potential—ΔµH^+^) (von Ballmoos et al. 2008, 2009; Junge et al. 2009; Kagawa 2010; Romanovsky and Tikhonov 2010; Tikhonov 2013a). The rotors of both the motors are constructively linked with each other and rotated jointly in opposite directions; the source of energy is either ATP (when dealing with ATP hydrolysis) or electrochemical potential—ΔµH^+^ (when dealing with ATP synthesis).

In 1993, Wolfgang Junge (see Junge 2013 and references therein) presented a physical model to explain torque generation by proton flow through F_0_. It was based on Brownian rotary fluctuations of the *c*-ring relative to subunit *a*, electrostatic constraints, and two non-co-linear access channels for the proton to the ion-binding residue in the middle of one leg of the hairpin-shaped *c*-subunit. According to Junge’s model, the interplay of random Brownian motion and directed electrochemical driving force (‘Langevin dynamics’) is a common feature of all nanomotors, as pioneered by Howard Berg’s model for the proton drive of bacterial flagella. But the current concept (Junge et al. 2009) describes this motor as the rotary electromotor, F_0_. Apparently, Junge et al. (2009) have in mind that this motor is a direct current electric motor since it is assumed that the rotation of the rotor occurs because of the energy from the proton flux (positive charge). At the same time, in the technical electric motors, only the electron energy is used to perform work. Miller et al. (2013) have assumed that ion-driven rotary motors are driven by an electric potential and ion gradient across a membrane, somewhat like a battery-powered electric motor. The imbalance of protons and potentials between the proton half-channels creates an electric dipole moment that generates electric field lines going around the *c*-ring and between channels. The field emanating from the half-channels can only generate a net torque. This proposed torque generation mechanism is based on the turnstile rotary mechanism of Vik and Antonio (1994), which is similar to Junge et al.’s model (see Junge et al. 2009), in which the *a*-subunit contains two offset half-channels allowing entry and exit of protons that bind to the rotor.

There is an important feature for the functioning of technical electric motors: All motors are built on the principle of repulsion with a complex system of windings to avoid “dead zone.” A direct electromotor or an alternating electric current motor built on the principle of attraction is still unknown. In order to avoid violation of the principles of electrodynamics, authors of F_0_ electric motor model have proposed the principle, which is based on the notion of Brownian motion. Besides, electric motors cannot function in the medium of electrolytes by short-circuiting. Thus, on the one hand, the model with rotation of *c*-ring as technical electric motor, in our opinion, is unrealistic, and on the other hand, it is unlikely that the chaotic Brownian motion with random fluctuations may play a crucial role in such ordered system as ATP synthase, wherein the rotor (*γ*-subunit) can rotate about 100 revolutions per second. At the same time in biological systems, electrochemical forces and hydrophobic interactions play an important role. It is well known that the electric potential is generated in closed membrane systems by electrochemical forces.

We find it difficult to accept the current model of the electric motor for F_0_ for reasons, listed below:

1. The electromagnetic induction is necessary to provide rotation of the electric motor and it is absent in this system (there are nanomotors acting by electrochemical forces without electromagnetic induction (Foroughi et al. 2011)).
2. The rotation of the *c*-ring is unlikely in membranes due to high resistance, since the protruding domains of *c*-monomers in the intermembrane space (the C-terminal and N-terminal portion) and the matrix (loops of hairpins) are formed from charged amino acids, which interact with aqueous solution.
3. There is no coupling between electric motor rotation and phosphate ions, although in the presence of ADP and Pi, the open probability (the probability of finding the channel in the open state) decreased, and the CF_0_ channel was blocked almost completely (Wagner et al. 1989).
4. There are data that contradict current model of the electric motor model: there is a question against rotation of F_0_c-ring (Tsunoda et al. 2001). Another work on binding of mAb molecule to F_0_c-ring (Deckers-Hebestreit et al. 2000) demonstrates the impossibility of rotation of the F_0_c-ring, because mAb molecules bind simultaneously to F_1_, polar loop in one or two *c*-monomers and the total large size (75 Å, molecular weight, 150 kDa) must interfere in the rotation of F_0_c-ring as well as *γ*-subunit by circular mode on loops of *c*-monomers (just four or five *c*-monomers participate in the interaction with *γε*-subcomplex). Moreover, the hypothetical two proton-conducting pathways (so-called “half-channels”) in the structure of F_0_ have not yet been observed (Gohlke et al. 2012), although such a proton pathway could exist.

Thus, we believe that the current picture of the «electromotor» for ATP synthase must be examined further. And, we present an alternate model: a hydrostatic model for the rotation of the *γ*-subunit associated with cyclic, low-amplitude changes in volumes of organelles that provide regulatory role in electron transfer.

Kasumov et al. (1991a, b, c, d) have already made the suggestion that cyclic shrinkage and swelling of mitochondria determine the electron and proton transfer and coupled phosphorylation of ADP. This means that shrinkage of organelles results in narrowing of the intracristal space, and a contact is established through the dimers of Cyt *bc*_1_ complex between the folds of internal mitochondrial membrane in the intermembrane space and through the Cyt *b*_6_ *f* complex of the flat thylakoid *bag* in chloroplasts that perform a regulatory role in the electron transfer chain (ETC). Thus, an electron from [2Fe–2S] cluster of one dimer is transferred to heme *f* of another dimer on the membrane of the opposite side and is then further transferred to the final acceptor. As a result of separation of charges, an electrochemical potential arises. The electrochemical potential, ΔµH^+^ (i.e., Δψ—electric potential plus ΔpH—proton gradient) is necessary for the vectorial transport of protons during ATP synthesis as well as transport of cations and anions that induce cyclic swelling–shrinkage of organelles. Lehninger (1966) had suggested that a part of energy (about 20 %) generated during the transfer of electrons is used in volume changes and these volume changes, in our opinion, in turn, promote further electron transfer along the chain that indicates interdependence of these processes and, thus, it is not a simple consequence of the volume change.

In this view, an obvious question arises:

What is the mechanism of phosphorylation in the ATP synthase, and how the swelling–shrinkage, which lasts for very short time periods (parts of milliseconds), is provided and what channels are involved in, if the membrane has limited permeability for ions?

## Comparison of the properties of ion channels and ATP synthase

It is known that oligomycin inhibits swelling and shrinkage as well as the activity of ATPase (Lehninger 1966), and oligomycin sensitivity-conferring protein is necessary for perfect binding of F_1_-subunit with F_0_-subunit (Racker 1979). Also, electron microscopy data show that low-amplitude changes of mitochondria volumes occur between matrix and intermembrane space (Hackenbrock 1966; Lehninger 1966; Harris et al. 1968). Then, we may conclude that swelling–shrinkage of intermembrane space as well as thylakoids in chloroplasts occurs through ATP synthase.

Thus, we suggest that ATP synthase is an ion pump-pore-oligoenzyme complex with a voltage sensor and gating mechanism. Here, in this picture, the *channel* is composed of two parts: one is the membrane-integrated (F_0_c), and the second a hexamer *α*_3_ *β*_3_. The gating mechanism, in our opinion, consists of alpha-helix regions of *γ*-subunit, *α* – helical loops of catalytic *β*-, and regulatory *α*-subunits, and this gating mechanism is linked with these ionic mechanisms. A blade of the pump, consisting of a globular part of *γ*-subunit and beta fold of *ε*-subunit, is located between channels near the *c*-ring entry.

The presence of glycine residues and hairpin transmembrane helices demonstrates a similarity of *c*-ring with a structure of potassium channel (Jiang et al. 2002). However, the selective potassium channel with narrowing part within the membrane is formed by four subunits, where *C*-terminal helix of monomer hairpin is located inside a tetramer and has an internal diameter of about 1.2 nm (Jiang et al. 2002). But, *c*-ring in the form of hourglass consists of 8 to 15 subunits (for different species) with N-terminal helix on the inner side of monomer and possesses an inner diameter of 2.7 nm in the narrow place for *c*_11_-ring (Pogoryelov et al. 2012). Seemingly, such a structure makes the *c*-ring similar to Na-channel, which is non-selective for cations and anions.

In the above picture, opening and closing of ion channels would proceed in fractions of milliseconds, and each open channel, under physiological conditions, may selectively allow passage of ions for ~200 ns (Hille 1981). These temporal characteristics of the functioning of ion channels may allow the implementation of cyclic swelling–shrinkage of mitochondria, which supports our assumptions on changes in the mitochondria through ion channels.

Further, a set of events between changing of potential and physiological reaction starts from motion, reorientation, or structural changes in the voltage sensor, which a molecule is able to react to changes in the potential. By moving or rotating inside the membrane (or inside protein subunits) under the influence of membrane potential changes, the voltage sensor produces electric current—current of displacement that may be registered under corresponding conditions (Almers 1981). We suggest that the *c*-ring in ATP synthase is similar to Na^+^-channel of more than 10 Å (about 20 Å) in diameter. Na^+^-channel is a pore (Armstrong 1981), and in the open state it is permeable to a large number of cations (Hille 1981), organic (e.g., ammonium, guanidium) as well as inorganic (Li^+^, Na^+^, K^+^) ions, and this allows ions to pass through the channel to be in contact with a large number of water molecules. Conformational changes in Na^+^-channels are regulated by electric field, within the membrane, as well as by certain chemical mediators. Here, Na^+^-channel would be closed in the resting state and open for ions in case of membrane depolarization from the initial potential of −75 mV to more positive potentials, than −55 mV (Hille 1981).

The *c*-ring channel is not a pure Na^+^-channel as is confirmed by the following data. Firstly, the inactivation of Na^+^-channel receives partly or fully the required voltage from the activation process; further, it does not need its own mobile charge (Armstrong 1981). Ion channel in ATP synthase, probably, is regulated by a mobile charge—proton that blocks ion channels (Hille 1981). Aspartic acid and glutamic acid residues at the c-ring in the middle of membrane may perform this role. Secondly, ouabain, an inhibitor of Na^+^-channel, does not influence mitochondria swelling–shrinkage (Lehninger 1966).

Unexpectedly, Ca^2+^ ions pass freely through Na^+^-pores (Armstrong 1981), which supports our earlier suggestion on this point (Kasumov et al. 2013b, d). Ca^2+^ ions induce swelling of energized mitochondria (Lehninger 1966). The presence of Ca^2+^ ions in the surrounding influences the gating process: changes in its concentration lead to the same result as changes in the membrane potential. This is explained by the fact that there is screening of fixed negative surface charges near the ion channels. Excess of Ca^2+^ ions, near the membrane surface, leads to generation of potential shift, which is registered by *voltage sensor* in the channel (Hille 1981). Tenfold decrease in the concentration of Ca^2+^ ions is approximately equal to depolarization of about 15 mV (Armstrong 1981), which highlights an important role for Ca^2+^ ions in the regulation of the gating mechanism, and in the maintenance of the composition of cations and anions in the organelles. Relatively high permeability for Ca^2+^ ions indicates that the pore diameter, on the average, should be substantially greater than 10 Å and Na^+^-pore, seemingly, is narrowed in one place only—in the middle of the membrane (Armstrong 1981) as seen in the structure of *c*–ring in ATP synthase.

Hille (1981) has suggested that two negative charges might form a complex on, e.g., an oxalate ion (^−^OOC–COO^−^) that chelates bivalent ions. The complex is saturated under physiological concentrations of Ca^2+^ ions. Protonation of the carboxyl group would lead to electrostatic blockage of the channel, i.e., the protonated acid forms a high-energy barrier, because it does not provide strong interaction of charges that are required for stabilization of partly dehydrated cations (Hille 1981). Such carboxyl groups in ATP synthase may be provided by Glu and Asp residues from the *α*—helical loops in *α*_3_ *β*_3_-subunits. Moreover, each *c*-subunit F_0_c-ring in *E. coli* contains an acidic residue (Asp 61) with carboxyl group that is available also on the lipid side in the middle of the membrane, and this residue is suggested to play an important role in proton translocation through protonation–deprotonation process (von Ballmoos et al. 2009). However, a molecular mechanism of proton translocation through F_0_ and coupled ATP synthesis/hydrolysis is still obscure (see e.g., Fillingame et al. 2000).

We note that data on conductivity of the channel in CF_0_-C_1_F_1_ are contradictory and, in our opinion, there are two different channels, a proton channel and non-selective channel for cations, and anions. Feniouk et al. (2004) have observed that dependence of the rate of H^+^ translocation (6,500 H^+^/s at 100 mV) through CF_0_ saturates at a specific level; this value is probably small for specialized proton channels. Wagner et al. (1989) reconstituted purified chloroplast ATP synthase (CF_0_-CF_1_) into asolectin bilayer membranes on the tip of a glass pipette and revealed a high channel unit conductance. It was shown that at membrane voltages <100 mV low open probability with concomitant open times in the µs range takes place. According to Wagner et al. (1989), these data suggest a gated mechanism with channel openings in the µs timescale (<100 µs) for energy coupling in the enzyme complex.

After removal of CF_1_ from the liposomes by NaBr treatment, CF_0_ was shown to display various kinds of channels that were also permeable to monovalent cations (Sconknecht et al. 1989; Wagner et al. 1989). The probability of the CF_0_-CF_1_ channel being open increased considerably with increasing membrane voltage, but in the presence of ADP and Pi, this probability decreased and the channel was almost completely blocked (Wagner et al. 1989). These data indicate the existence of two different channels for protons and cations, as well as a gating mechanism in ATP synthase, where F_1_ plays a specific role. On the one hand, F_1_ is itself a channel, and on the other hand, it opens and closes the channel in F_0_.

It is believed that the number of *c*-monomers characterizes H^+^/ATP ratios (Ferguson 2000, 2010; von Ballmoos et al. 2009; Walker 2013) since each 360° rotation of the rotor of the ATP synthase produces three ATP molecules, and each 360° rotation of the *c*-ring requires the translocation of the same number of protons as the number of *c*-subunits in the ring. Consequently, the proton-to-ATP ratio of an ATP synthase can be calculated by dividing the number of *c*-subunits by three (von Ballmoos et al. 2009; Walker 2013). In this case, the *c*-ring as a rotor with a large diameter (e.g., chloroplasts) will be held in the 360° full circle for a longer time as compared with a *c*-ring having a smaller diameter (e.g., mitochondria), and hence, the efficiency of ATP synthesis in ATP synthases with the large diameter of *c*-rings per unit of time will be less, although, in our opinion, the efficiency of ATP synthesis in chloroplasts per unit of time must be greater than in mitochondria.

Based on the above, and other data in the literature, we have already suggested a mechano-chemiosmotic model of coupling of electron transfer to ATP synthesis, where the electron transfer along electron transfer chain, proton transfer, transport of cations, low-amplitude swelling–shrinkage, and ATP synthesis are coupled processes (Kasumov et al. 2011, 2012, 2013a, b, c, d). This model indeed uses the chemiosmotic model of Peter Mitchell (see Mitchell 1961, 1966) as its basis, and supplements it by dynamic properties characteristic of biological structures with particular attention to a regulatory role of a low-amplitude swelling–shrinkage of organelles. As noted above, Lehninger (1966) had already suggested such a relationship.

We will attempt to explain below the mechanism of *γ*-subunit rotation taking into account the peculiarities of twisted carbon nanotube nanomotor rotation, stabilization–destabilization of protein molecules by polyvalent anions–cations, principles of ion channel functioning, complex structure of ATP synthase, including the γ-subunit, and a complex interdependence of processes coupled in ATP synthesis with swelling–shrinkage, as suggested earlier (Kasumov et al. 2011, 2012, 2013a, 2013b, 2013c, 2013d). In our opinion, such a mechanism of ATP synthesis occurs in vivo and may not be compatible with some of the data obtained in vitro, where the rate of ATP synthesis is relatively very low.

## Mechano-chemiosmotic model

**Fig. 3:**
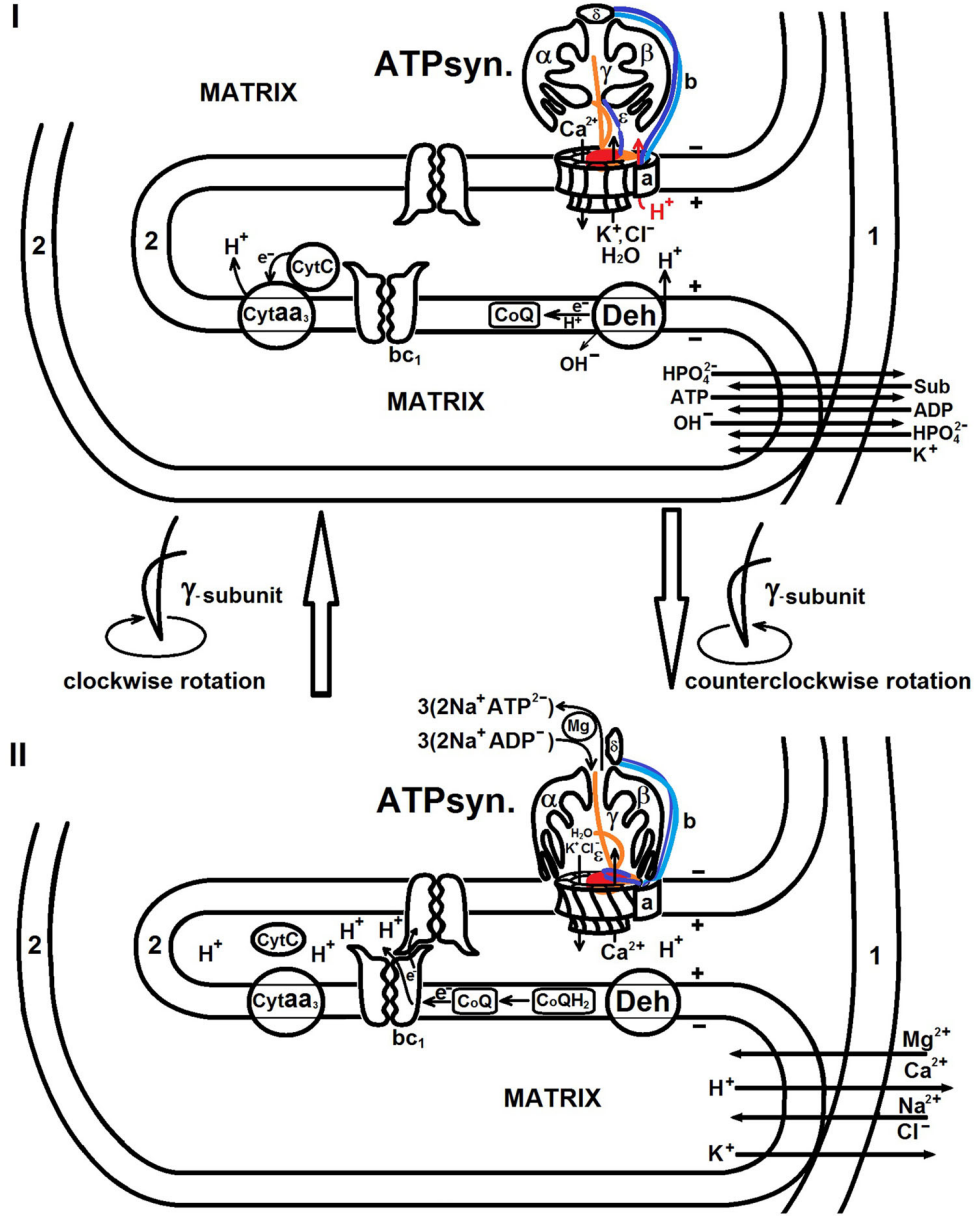
A cyclic swelling (I) and shrinkage (II) of mitochondria intracristal space associated with the transfer of electrons through the cytochrome *bc*_1_ dimers, ion transport in ATP synthase and ATP synthesis; electron transfer from dehydrogenase (Deh) to cytochrome oxidase (Cyt*aa*_3_) through ubiquinol (CoQH_2_), dimeric cytochrome *bc*_1_ and cytochrome *c*, *1* outer membrane, *2* inner membrane, *α*, *β*, *γ*, *δ*, *ε*, *b*_2_, *a*—subunits of ATP synthase. Schematically shown is the rotation of the γ-subunit counterclockwise direction during energization and clockwise direction in the synthesis of ATP—deenergization. This figure was made by the authors on the basis of drawings from Lehninger (1966)

We present below a model of mechano-chemiosmotic coupling; we have arbitrarily divided its description into two stages that take into account rotation of *γ*-subunit and the coupled processes, including cyclic low-amplitude swelling–shrinkage of mitochondria *intracristal* space (Fig. 3).

### Intracristal space shrinkage stage (Fig. 3, I)

A scenario follows. Intracristal space is in the swelling state. Synthesis of ATP has been completed and all newly synthesized ATP molecules are tightly bound to the active centers of ATP synthase. The *α*_3_ *β*_3_-hexamer is located at a distance of ~3 nm from the membrane. Protons that had earlier penetrated into the matrix, due to proton gradient, are driven out from the matrix to the cytoplasm, putatively, in antiport fashion with cations, including calcium ions. Reduced cytochrome *c* transfers an electron to cytochrome oxidase—Cyt *aa*_3_. Dehydrogenase (Deh) removes protons and electrons from a substrate. One proton (H^+^) is transported to the intracristal space, and an electron is transferred to ubiquinone followed by formation of ubisemiquinone. The latter, while being reduced to ubiquinol, seemingly, promotes formation and transfer of one OH^–^ group. Matrix pH acquires alkaline character at pH 7.6. Thus, a pH difference (ΔpH) between the two sides of the inner membrane appears. There are always reserves of phosphate in mitochondria in the form of calcium phosphate; nevertheless, consumption of phosphate ions takes place. At this stage, OH^−^ from the matrix is exchanged for phosphate ion from the surrounding. Because C-terminus of *ε*-subunit is in extended conformation and it protonates *β*_1_-subunit, the proton channel in the region of the contact of *a*- and *b*_2_-subunits with *c*-ring (von Ballmoos et al. 2009) in the matrix opens and protons penetrate the matrix through the proton channel utilizing proton gradient. Ion pump of ATP synthase pumps calcium ions into intracristal space through the ion channel (*c*-ring) of ATP synthase and K^+^ and Cl^−^ ions with hydration shell into matrix. Cl^−^ anions are counterions for protons in order to complete the electrical circuit through the membrane; after this step, shrinkage of the intracristal space takes place. Pumping of calcium (Ca^2+^) ions into the intermembrane space is an energy-dependent process, and it proceeds under polarization of the membrane. Older data on calcium ions accumulation in mitochondria (Vasington and Murphy 1962) and submitochondrial particles (Vasington 1963) provide evidence for this conclusion. This would mean that calcium cations are pumped into the intermembrane space across matrix.

Thus, in this picture, a part of energy of electrochemical potential, about 20 % (Lehninger 1966) is used for transport of hydrated cations. It is necessary to take into account the fact that channel opening is a potential-dependent process and, seemingly, needs additional protons. This stage corresponds to a state of cyclic conformational change in mitochondria: substrate-induced energization of inner membrane occurs, as was described by Harris et al. (1968).

**Fig. 4:**
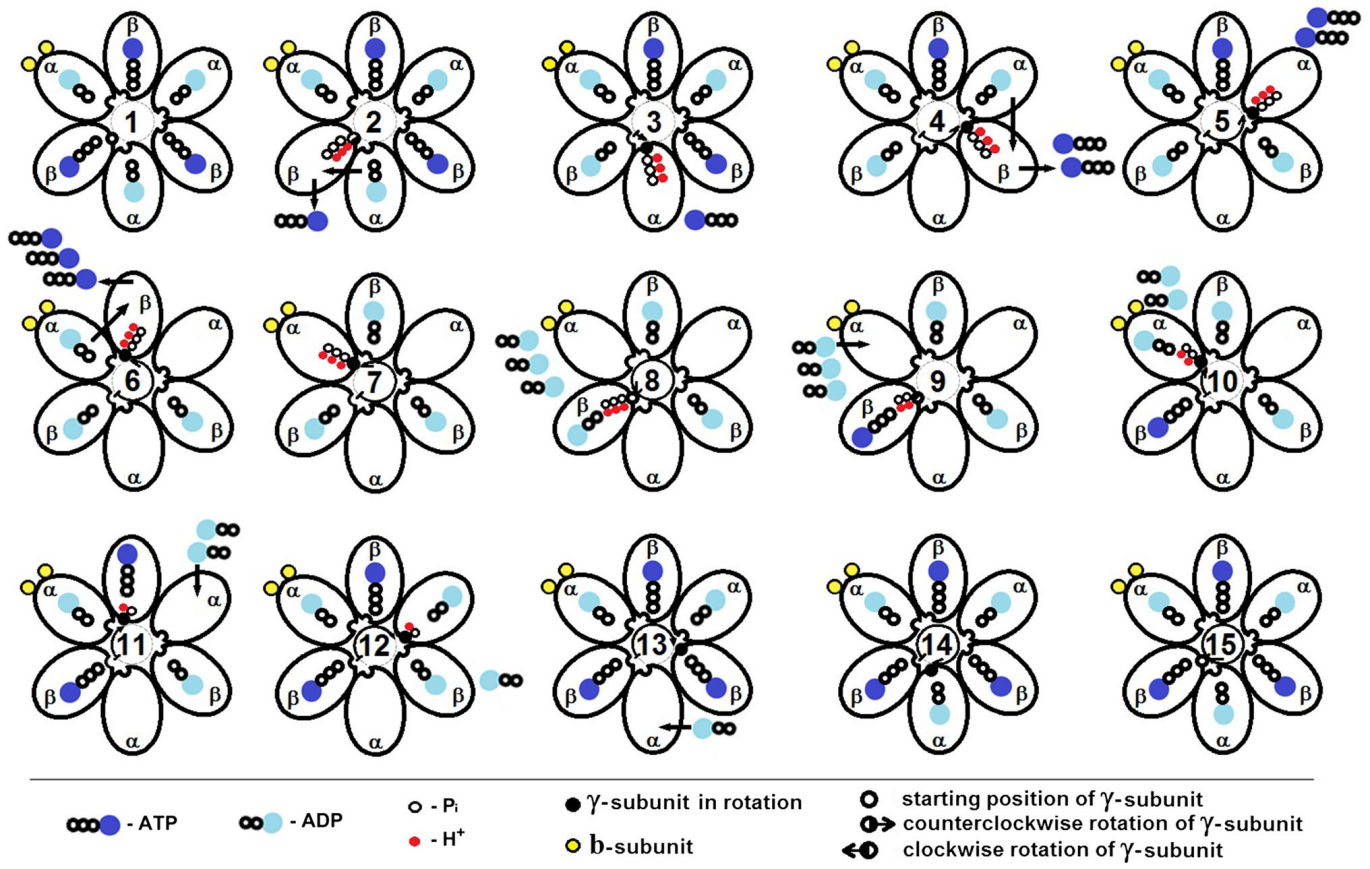
A rotary mechanism of the ATP synthesis, the release of ATP, and the loading of ADP. Arrows indicate the ATP release from the active site of *β*-subunit and the loading of ADP to the active site of *α*-subunit; the transition of ADP from the active center of *α*-subunit to active site of *β*-subunit. The *arrows* in the center of the figure show the rotation of the *γ*-subunit in steps of 30° and 90° counterclockwise direction and clockwise direction. *1 γ*-subunit is in the starting position near the *β*-subunit. ADP and ATP are tightly bound in the *α*-subunits and *β*-subunits, respectively; *2* C-terminal of *γ*-subunit connects three protons and three phosphate ions during the energization process; the active center is opened and ATP molecule is released due to electrostatic repulsion from the active center and moves to the top of the *α*_3_ *β*_3_-hexamer; *3* *γ*-subunit starts to rotate counterclockwise direction and contacts the alpha subunit; and ADP moves from alpha subunit to the beta subunit; *4*–*8* γ-subunit continues to rotate counterclockwise direction and stops at the beta subunit, making 360°; at this time, all the molecules of ATP are in the upper part of the *α*_3_ *β*_3_-hexamer, and all ADP molecules are passed on to the beta subunits from the alpha subunits; and F_1_ is attracted to the membrane; *8*, *9* three molecules of ATP in upper part of *α*_3_ *β*_3_-hexamer are exchanged with three molecules of ADP from the matrix in the presence of sodium ions during full energization of the system; *9* after full energization, the first molecule of ATP is synthesized in the *β*-subunit at the starting position of γ-subunit; *10 γ*-subunit starts to rotate clockwise direction and contacts with the *α*-subunit. ADP molecule moves from the upper part of the *α*_3_ *β*_3_-hexamer into *α*-subunit; *11*–*15* *γ*-subunit continues to rotate clockwise direction and stops at the *β*-subunit, making 360°; at this time, three ATP molecules are synthesized and remain tightly bound on the *β*-subunits; at the same time, ADP molecules are bound on the alpha subunits; and F_1_ is spaced from the membrane during complete deenergization

Pumping of calcium ions into intracristal space decreases the buffer capacity of the matrix and protons shift pH value in the matrix from 7.6 to 7.0 (value of 7.6 is a preliminary estimate; it is necessary to measure the precise value of the matrix pH). At pH 7.0, negative and positive amino acid residues become ionized. Protonation takes place with subsequent binding of phosphate ions of positively charged residues at the expense of neighboring negatively charged residues of the *b*_2_-subunits. We suggest that at this moment, two positively charged residues (lysine or arginine) at the N-terminal part and three positively charged residues at the C-terminal part of *γ*-subunit contact the *α*-helical “DELSEED” loop (this loop is formed from negative amino acid residues: d-aspartic acid, E-*glutamic acid*) in *β*_1_-subunit, deprotonate it and shift the loop into its open state. In this open state, the synthesized ATP in the previous cycle is released due to its negative charge being repulsed (ATP carries a negative charge) by negative charges of phosphate ions at *γ*-subunit. Residues of the N-terminal part of *γ*-subunit acquire positive charge in parallel with residues of *b*_2_-subunits. Binding of phosphate ions to helically twisted *b*_2_-subunits is expected to promote further twisting of *b*_2_-subunits to stabilize the protein in the electric field that will cause the hexamer *α*_3_ *β*_3_ to approach the membrane. Phosphate binding on the N-terminal part of the *γ*-subunit and alpha-helix stabilization is expected to force the subunit to rotate counterclockwise direction in the electric field (transmembrane potential) due to its constructive feature (screw-like structure). A rotary mechanism of the ATP synthesis, the release of ATP, and the load of ADP are shown in Fig. 4.

Stabilization of the protein molecule through compression is suggested to proceed in the presence of monovalent cations (in this case, potassium ions) and polyvalent anions (phosphate ions). Possibly, there is dependence of angle rotation of the *γ*-subunit on the number of positive residues on the N-terminus domain of *γ*-subunit given its arc-shaped structure. Thus, rotation at 30° will bring the N-terminal part of the *γ*-subunit to the loop of *α*-subunit at the site of catalytic activity of *α*- and *β*-subunits. *α*-subunit protonates additionally two positive residues in the N-terminal part of *γ*-subunit and transits *α*-subunit to the open state, from where, an ADP molecule moves to the catalytic center of *β*_1_-subunit. In “non-catalytic” sites, the rate of nucleotide exchange is about two orders of magnitude lower than the catalysis rate, even with an energized membrane (Malyan 2007).

Since *α*_3_ *β*_3_-hexamer is attracted to the membrane due to twisting of *b*_2_-subunits in the counterclockwise direction (as a right helix-twisted cord attached to membrane and in its apical part), then, due to the screw-shaped structure of N-terminal domain of *γ*-subunit, its positively charged residues will be opposite to *α*-helical loops of *α*- and *β*-subunits in rotation of *γ*-subunit. Thus, rotation (Fig. 4, 9–15) of *γ*-subunit to 360° with stops at 30°, and at 90° (in our opinion, one phosphate ion twists a protein by 30°; the angle of rotation is determined by the angle between the nucleotide-binding sites of alpha and beta subunits of ATP synthase) will be sufficient for allowing N-terminal part of *γ*-subunit to contact *α*-helical loops of all *α*- and *β*-subunits according to their turn and to stop at *β*_1_-subunit, from which rotation is started. At the same time, *α*_3_ *β*_3_-hexamer will be dragged fully by *b*_2_-subunit to the membrane. By rotation of the *γ*-subunit, negatively charged ATP molecules are moved due to electrostatic repulsion by negatively charged phosphate ions from the *β*-subunits at the top of the hexamer, and ADP molecules move from *α*-subunits in free *β*-subunits. At full energization, three ATP molecules are released into matrix from the top of the hexamer of ATP synthase and three molecules of ADP are moved into hexamer from the matrix. We believe that sodium ions are involved in the loading of ADP and in the release of ATP from the top of the *α*_3_ *β*_3_-hexamer of the ATP synthase.

During proton transport from intermembrane space to matrix, shrinkage of intracristal space and a shift of pH value up to 7.0 take place in the intracristal space. Dimers of Cyt *bc*_1_ complexes on the opposite sides of the membrane are asymmetrically contacted during shrinkage. This contact takes place due to electrostatic interactions of negatively and positively charged amino acid residues which are localized in big and small domains of cytochrome *c*_1_. These conditions are favorable for heme reduction of Cyt *c*_1_, because this heme is reduced maximally at pH >7 (Ma et al. 2008). Moreover, during this time, opposite dimers of cytochrome *bc*_1_ complexes come into contact with each other, and active centers of cytochromes *c*_1_ and [2Fe–2S]-clusters become close to each other.

In this picture, the shrinkage stage takes place, in our opinion, in about 8 ms. This stage corresponds to a state of cyclic conformational change of mitochondria: phosphate-induced conversion of energized membrane into energized twisted state of inner membrane, as described by Harris et al. (1968).

### Intracristal space swelling stage (Fig. 3, II)

If ADP is added to mitochondria that are in energized and twisted state of inner membrane, then ATP is synthesized; and mitochondria swell and transit to deenergized state (during addition of calcium ions, mitochondria simply are deenergized with subsequent depolarization and become swollen) (Harris et al. 1968). ADP causes depolarization of the membrane, and ion channel in *c*-ring is opened and the ion pump at this stage works in the reverse mode, i.e., it pumps out calcium ions toward lower part of hexamer, and potassium and chloride ions into intracristal space that undergoes swelling. The swelling stage (Fig. 4, 1–8) must be very fast and it must take, in our opinion, about 1 ms. Because calcium ions cause destabilization of protein molecule due to binding of phosphate ions, then dephosphatising of *γ*-subunit and *b*_2_-subunits takes place. Under the condition of depolarization, and according to the principle of carbon nanomotor functioning (Foroughi et al. 2011), *γ*-subunit will rotate in clockwise direction. During *γ*-subunit rotation (Fig. 4, 9–15), three molecules of ADP will move to *α*-subunits from the top of the hexamer, *α*-helical loops of *α*- and *β*-subunits will be protonated, and the enzyme will transit into the closed state. In this case, ATP molecules will be synthesized and remain tightly attached. They will be released only when they will be under conditions of energization in the next cycle. Cytochrome *c* is reduced receiving electrons from heme *c*_1_ and the cycle repeats again. Here, ATP synthase will look like a mushroom on the membrane with the length of its leg to be ~3 nm. Indeed, direct evidence for this process is the restructuring of the F_1_–F_0_ complex of submitochondrial particles under the influence of sound waves (Syroeshkin et al. 1998). In the presence of phosphate ions, F_1_-complex is immersed into the membrane, while adding ADP to such submitochondrial particles allows one to see the leg connecting F_1_ and F_0_ complexes (Syroeshkin et al. 1998). Moreover, the existence of such conformational states of the ATP synthase is consistent with the hypothesis of Boyer (1989) and of Galkin and Vinogradov (1999) about different conformational states of the enzyme participating in ATP synthesis or hydrolysis.

It is necessary to note that researchers have obtained contradictory results concerning the position of ATP synthase in the membrane: it looks like a mushroom or its *β*-subunit is in direct contact with phospholipids (Skulachev 1989). Because during energization of membrane, the *α*_3_ *β*_3_-hexamer is attracted to the membrane and its *β*-subunit is in direct contact with phospholipids. The deenergization of membrane leads to a separation of *α*_3_ *β*_3_-hexamer from the membrane, and it protrudes ~3 nm toward the matrix, where ATP synthase looks like a mushroom with gamma subunit as a leg of the mushroom.

In mitochondria with high-amplitude swelling, electron transport is stopped and ATP is required to return it to its normal state (Lehninger 1959). Rotation of *γ*-subunit during ATP hydrolysis will be as in the energization process, but protons and phosphate ions will be produced during ATP cleavage only.

Lohrasebi and Feshanjerdi (2012) have stated that F_0_ is the ion pump as opposed to the dominant opinion that the *c*-ring is the rotor; they have even built a pump, which is capable of producing a gradient of ions. It has a blade, which pumps ions from one compartment to another. Operation of this pump is similar to the principle of *c*-ring as ion channel. Many experimental facts agree with the mechanism presented by our model; some of these are listed below:

- Tributyltin chloride, a special inhibitor of ion access through subunit *a*, inhibits rotation of F_0_ motor (as currently many assume that there is an F_0_ electromotor and its rotation occurs due to protons (von Ballmoos et al. 2008, 2009; Junge et al. 2009; Kagawa 2010; Romanovsky and Tikhonov 2010; Tikhonov 2013a) by 96 % (Ueno et al. 2005; see also von Ballmoos et al. 2009).
- Transport of anions (for example, chloride and bicarbonate), together with potassium ions, was proved by the following results. First, 100 water molecules leave mitochondria during hydrolysis of one ATP molecule (Lehninger 1966), if the secondary hydrate shell is equal to 45 for potassium and 55 for chloride ions, then the result is 100 water molecules. Second, ATP synthesis depends strongly on anions and it is suggested that there is a separate anion channel that differs from proton channel (Agarwal 2011). Moreover, tributyltin is a blocking agent of anion channel. These facts, on the one hand, allow us to understand the mechanism of mitochondrial swelling–shrinkage due to the movement of ions, but and on the other hand, support our hypothesis about the mechanism of hydrostatic nature of *γ*-subunit rotation. Since, in the synthesis of ATP movements of protons, chloride, calcium, potassium ions through the membrane of mitochondria are interconnected and rotation of the gamma subunit occurs with their participation, as described above, then the disruption of movement of ions should block rotation of the gamma subunit.
- Replacement of *γ*Met23 by Arg or Lys disrupts ATP-dependent proton transfer and ATP synthesis (Sambongi et al. 2000). N-terminal domain of the γ-subunit constitutes the structure in the supercoiled form of an arc relative to the C-terminal domain. Amino acid residue γMet23 connects these two domains at the bottom and γMet246 at the top portion of arc. The presence of such a structure is one of the important conditions for rotation of the γ-subunit by mechano-chemiosmotic model. Consequently, in the case of disruption of the linkage between them, the N-terminal part of *γ*-subunit cannot contact alpha-helical loops of *α*- and *β*-subunits. On the other hand, an addition of one more positive charge at the N-terminal part of *γ*-subunit will not allow the contact of *γ*-subunit and alpha-helical loops of *α*- and *β*-subunits and, as a result, it will break the rotation of *γ*-subunit. So, two central *α*-helices of *γ*-subunit, indirectly regulate the activity of F_1_-ATPase in cyanobacteria (Sunamura et al. 2012) and their conformational changes will affect the activity of ATP synthase. For example, removal of 8 and 20 N-terminal amino acid residues preserves hydrolytic activity up to 30 and 6 % (Ni et al. 2005), which indicates an important role of N-terminal helix. At the same time, removal of 20 C-terminal amino acid residues of *γ*-subunit weakly influences its activity (Sokolov et al. 1999), because this area of *γ*-subunit is not too important for the rotation of *γ*-subunit, which is consistent with our mechano-chemiosmotic model.
- The experiment of Wächter et al. (2011) confirms one of the suggestions of our model, which states that *b*_2_-subunits are twisted during the functioning of the ATP synthase. If the authors of the experiment had put tags in the field of dimerization domain (residues 53–122) of *b*-subunit to contain a two-stranded right-handed coiled coil with offset helices (Wood and Dunn, 2007), we predict that they would have found a much greater angle of rotation (the total twisting, due to the positively charged residues of the dimerization domain is greater than 1,000°, i.e., 34 residues × 30° = 1,020°), compared to the one observed (40°). A similar principle of operation by twisting of the filament is shown for bipolar assembly domain that directs four Kinesin-5 subunits to form a bipolar minifilament (Scholey et al. 2014). Bipolar assembly is a novel 26-nm four-helix bundle, consisting of two antiparallel coiled coils at its center, stabilized by alternating hydrophobic and ionic four-helical interfaces, which based on mutagenesis experiments, are critical for tetramerization.
- Watanabe et al. (2014) investigated the role of three charged residues of F_1_ (the p-loop lysine in the phosphate-binding-loop, GXXXXGK(T/S), a glutamic acid that activates water molecules for nucleophilic attack on the γ phosphate of ATP (general base), and an arginine directly contacting the γ phosphate (arginine-finger) in catalysis and torque generation by analyzing alanine-substituted mutants in the single-molecule rotation assay. It was shown that, although these charged residues contribute to highly efficient catalysis, they are not indispensable to chemo-mechanical energy coupling, and the rotary catalysis mechanism of F_1_ is far more robust than previously thought.
- The *γ*-subunit rotation mechanism is consistent with the torsional mechanism of energy transduction (Nath 2008) and takes into account the contribution of importance of the water entropy effect (Yoshidome et al 2011). We believe that the data of Itoh et al. (2004) do not contradict our model, since the rotation of the gamma subunit is needed for the delivery of protons and phosphate ions to the active centers. In fact, this rotation can be caused by electrochemical interactions, mechanical action (Itoh et al. 2004), or ultrasound (Syroeshkin et al. 1998), regardless of the source of energy.
- The *c*-subunit of the mitochondrial F_1_-F_0_ATP synthase, which is located on all surfaces of inner membrane, has been recently found to be a fundamental component of the mitochondrial permeability transition pore –mPTP (Bonora et al. 2013). Alavian et al. (2014) have shown that the purified reconstituted *c*-subunit ring of the F_0_ of the F_1_F_0_ATP synthase forms a voltage-sensitive channel, the persistent opening of which leads to rapid and uncontrolled depolarization of the inner mitochondrial membrane in cells. Prolonged high matrix Ca^2+^ enlarges the *c*-subunit ring and unhooks it from cyclophilin D/cyclosporine A binding site in the ATP synthase F_1_ provides a mechanism for mitochondrial permeability transition pore, i.e., for mPTP opening.

## Concluding remarks

The mechano-chemiosmotic model, discussed in this paper, combines fundamentals of chemical, conformational, and chemiosmotic theories of coupling of electron transport to ATP synthesis in energy-generating membranes, and responds to most difficult questions in the area of bioenergetics. H^+^/ATP ratio and differences in diameters of *c*-rings in mitochondria, chloroplasts, and bacteria are among such questions (Walker 2013), although it is believed that the H^+^/ATP ratio is directly related to the number of *c*-monomers (Ferguson 2000; 2010). According to the mechano-chemiosmotic model, differences in diameters of *c*-rings in ATP synthases in mitochondria, chloroplasts, and bacteria are linked to the fact that a number of *c*-rings in chloroplasts and bacteria are reduced in comparison with that in mitochondria and rings of larger diameters are required to pump stroma or cytosol volumes during short time period (see Appendix for a discussion on the diameter of the *c*-ring).

Direct determination of the H^+^/ATP ratio is difficult. One approach is to compare the magnitude of the Δ*G* for ATP synthesis with the pmf. Steigmiller et al. (2008) found that H^+^/ATP ratio of 4 for both the enzymes, which is inconsistent with the *c*-subunit stoichiometries of 14 and 10 for thylakoids and *E. coli*, respectively. At present, most investigators are inclined to believe the H^+^/ATP ratio deduced from the *c*-subunit stoichiometry and to assume that the thermodynamic measurements must have a yet-to-be-identified flaw (Ferguson 2010).

The H^+^/ATP ratio may be estimated according to mechano-chemiosmotic model as follows:

H^+^/ATP = (*n*H^+ ^+ 3H^+^)/3ATP, where *n*H^+^ is the number of protons required for the pH change in the matrix of mitochondria, stroma of chloroplasts, or cytosol of bacteria to neutral pH value of 7.0; 3H^+^ is the number of protons required for the synthesis of three ATP molecules at active sites of the enzyme. In chloroplast stroma, pH is equal to 7.8–8.0 (Tikhonov 2013a). If the mean pH value is equal to 7.9, then H^+^/ATP = (9H^+ ^+ 3H^+^)/3ATP = 4 H^+^/ATP ratio for chloroplasts, which is in agreement with the literature data of H^+^/ATP ratios of 3.9 ± 0.2 at pH 8.45, and 4.0 ± 0.3 at pH 8.05 (Turina et al. 2003; Steigmiller et al. 2008).

Mitochondrial pH and pH gradient decrease during cytosolic Ca^2+^ elevation and individual mitochondria undergo spontaneous alkalization transients (Poburko et al. 2011; Santo-Domingo et al. 2013). Calcium ions perform a regulatory role in mitochondria and in cells. Increases in the free calcium ion concentration in the mitochondrial matrix activate mitochondrial dehydrogenases to stimulate oxidative phosphorylation (Hajnóczky et al. 1995), whereas Ca^2+^ increases in the intermembrane space stimulate the uptake of substrates of oxidative phosphorylation (Satrústegui et al. 2007; Contreras et al. 2007). Thus, these data indicate that the calcium ions are moved cyclically between the external environment, the matrix, and intermembrane space activating various processes.

The mechano-chemiosmotic model is based on the directed electrochemical driving force and the direct proof of this model is the acting nanomotor of carbon nanotube, which operates on the same principles. However, according to the principles of the electric motor model of Wolfgang Junge, it does not yet create the acting engine. Furthermore, it did not prove the rotation of *c*-ring by protons without ATP and other phosphate compounds. For this it is necessary to reconstitute a purified *c*-ring, *a*-, *b*-subunits complex in proteoliposomes, or in the membrane between the two compartments for the observation of the rotation. The rotation of *c*-ring can be observed (if the electric motor model is correct) by changing the pH to the acidic value, below pH 6 in the compartment of C-and N-terminal region of the *c*-monomers (P side) and to the alkaline values in the other compartment, through the method of acid/base transition experiments (see e.g., Jagendorf and Uribe 1966; Wagner et al. 1989; von Ballmoos et al. 2009).

During ATP synthesis, energy is, predominantly, used first, for the delivery of phosphate ions and protons to the *α*_3_ *β*_3_-hexamer against the energy barrier with the help of C-terminal alpha-helix of *γ*-subunit that acts as a lift; second, for the formation of phosphoryl groups; and third, for the release of ATP molecules from the active center of the enzyme and the loading of ADP. We suggest that sodium ions are involved in the loading of ADP and in the release of ATP from the top of the *α*_3_ *β*_3_-hexamer of the ATP synthase.

According to the mechano-chemiosmotic model, during energization of the membrane by either electron transfer or ATP hydrolysis, the *γ*-subunit rotates in the counterclockwise direction regardless of the energy source. ATP synthesis causes the deenergization of the membrane, where the γ-subunit rotates in clockwise direction. In order to synthesize ATP first, the energization of the membrane occurs in vivo; therefore, the *γ*-subunit must first rotate counterclockwise 360° during energization. This stage is absent in the current model based on the in vitro experiments, where Fo part of ATP synthase is “detached” from the membrane and *γ*-subunit rotates clockwise in the synthesis of ATP, and counterclockwise in the hydrolysis of ATP, which is one of main weaknesses of the current model.

**Scheme 1:**
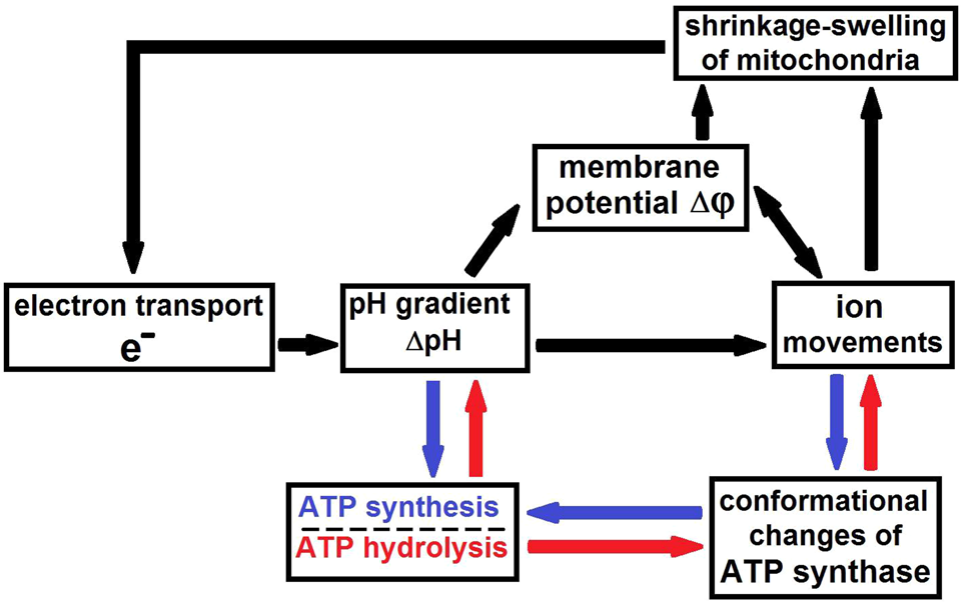
A schematic representation of the coupling of electron transport, ATP synthesis, ATP hydrolysis, movement of ions and low-amplitude shrinkage–swelling of mitochondria, thylakoids, and bacteria. *Black arrows* represent the general sites of coupling both in the ATP synthesis and in the ATP hydrolysis. *Blue* and *red arrows* represent the sites of coupling in the ATP synthesis and in the ATP hydrolysis, respectively

Thus, the pH gradient and membrane potential together lead to the formation of ATP according to the mechano-chemiosmotic model, which is in agreement with the Mitchell theory (Mitchell 1961); further, the mechano-chemiosmotic model provides further details on how the process occurs. The pH gradient creates not only a movement of protons due to pmf, which leads to a change in pH of the matrix, but it also initiates movement of cations; this movement of cations causes a shrinkage of mitochondrial intermembrane (intracristal) space. This shrinkage, in turn, is suggested to create a condition for electron transfer from [2Fe–2S]-cluster to heme *c*_1_. Thus, based on the suggestion of Lehninger (1966), coupling of electron transfer to the ATP synthesis occurs through shrinkage–swelling of mitochondria and depends on the presence of membrane potential, ADP, HPO_4_^2−^, Mg^2+^, K^+^, Na^+^, Ca^2+^, Cl^−^—ions, electron and proton transfers, and cyclic low-amplitude changes in the volume of mitochondria (see Scheme 1). We assume that mechanisms of ATP synthesis for chloroplasts, mitochondria, and bacteria are identical, where a role of calcium ions is very important. A Ca^2+^/H^+^ antiport actively moves Ca^2+^ from the stroma into the thylakoid lumen in the light and thylakoids are capable of accumulating approximately 2 nmol Ca^2+^ min^−1^ mg^−1^ chlorophyll from external concentrations of 15 μM (Ettinger et al. 1999).

Finally, a comparative analysis of the current mechanism of ATP synthesis according to Paul Boyer and mechano-chemiosmotic model of this paper is presented in Table 1.

**Table 1:** A comparison of Boyer’s model with mechano-chemiosmotic model

	Boyer’s model (Skulachev 1989; Boyer 1997; Tikhonov 2013a; Walker 2013)	Mechano-chemiosmotic model
Proton delivery	Proton is not delivered to the active center	Three protons are delivered to the active centers at *C*-terminal part of *γ*-subunit
What is the purpose of protons?	Protons are necessary for rotation of a rotor—*c*-ring, and *c*-ring rotates together with *γ*-subunit	Protons are necessary to change pH value in the matrix, stroma, or cytosol to pH 7.0 to provide negative and positive groups in the proteins
What is the major force that drives ATP synthesis in the ATP synthase functioning?	pmf is what drives ATP synthase. The force that causes a movement of the «rotor» of ATP synthase arises as a result of difference of potentials between outer and inner sides of the membrane (>220 mV) and is provided by proton flow, passing through a special channel in F_0_ located between subunits *a* and *c*	The major force for ATP synthase functioning is a proton gradient and membrane potential as is the case for the other model. Proton flow, passing through proton channel in F_0_ located at the border between *a*-, *c*- and *b*_2_-subunits, change pH of matrix, stroma, or cytosol to pH 7.0, and three protons are delivered to active centers. At the expense of proton gradient, transport of cations, and change of volumes of organelles take place. Membrane potential causes rotation of *γ*-subunit and twisting of *b*_2_-subunits. At the expense of membrane potential, three protons, three phosphate ions are delivered to active centers. Due to electrostatic interactions nucleophilic substitution takes place, phosphoryl groups are formed, three molecules of ATP are synthesized, ATPs are released from the enzyme, and ADP molecules are loaded to active centers
Does ATP synthesis require energy?	Energy is not required for ATP synthesis, but for its release (see below)	Energy is required for the production of phosphoryl groups
Does delivery of ADP and Pi to active center require energy?	Energy is required for delivery of ADP and Pi from the water phase to the active center. This is provided by mechanical movement of the side surface of *α*- and *β*-subunits during rotation of a «rotor»—*γ*-subunit, where conformational changes take place in catalytic centers	Energy is necessary to deliver ADP and Pi from the water phase to active center. ADP enters through the apical part of the hexamer during opening of the «lid-cap» of the hexamer—*δ*-subunit. Three Pi together with three protons are delivered to active centers at *C*-terminal part of *γ*-subunit
Does ATP release from the enzyme require energy?	Energy is required for the release of ATP from the enzyme. This is achieved by mechanical movement of the side surface of *α*- and *β*-subunits during rotation of the «rotor»—*γ*-subunit, where conformational changes take place in catalytic centers	Energy is required for ATP release from the enzyme. Under energization of membrane, *γ*-subunit rotates counterclockwise direction and negatively charged phosphate ions electrostatically repulse negatively charged ATP molecules to the apical apart of the hexamer. In case of full energization of the membrane, the «lid-cap» of the hexamer—*δ*-subunit opens and ATP molecules from the apical part of the hexamer are exchanged for external molecules of ADP with participation of sodium ions
H^+^/ATP ratio	H^+^/ATP ratio is linked to the amount of *c*-monomers in the *c*-ring	H^+^/ATP ratio is linked to the amount of protons (3 protons) required for the synthesis of ATP molecules and pH changes to neutral pH of 7.0
Low-amplitude changes of volumes of organelles	Low-amplitude changes of organelle volume are consequences and do not carry any function	Low-amplitude changes of volumes of organelles proceed as a result of membrane protonation and transport of cations. During shrinkage of the intermembrane space or thylakoids, electron transfer from ISP protein to cytochrome *c*_1_ or *f* takes place
Transport of ions—cations and anions	Transport of cations or anions is not meaningful and it is a consequence of a response to protonation of the membrane	Transport of cations and anions is active part of the mechanism, energy-dependent process at the expense of proton gradient. It changes the buffer capacity of matrix, stroma, or cytosol. Monovalent cations together with polyvalent anions participate in stabilization, but polyvalent cations (calcium), on the contrary, cause destabilization of protein molecules
Opening of active center of enzyme	Active center of the enzyme is opened by mechanical movement of the side surface of *α*- and *β*-subunits with the assistance of *γ*-subunit	Active center of the enzyme is opened by deprotonation of alpha-helical loops of *α*- and *β*-subunits by *γ*-subunit
Rotation of *γ*-subunit	*γ*-Subunit rotates due to rotation of the *c*-ring or in ATP hydrolysis	*γ*-Subunit rotates in the case of protonation and binding of phosphate ions to N-terminal subunit in the electric field
Role of *δ*-subunit	*δ*-subunit is a part of stator	*δ*-Subunit is a “lid-cap” of hexamer through which ADP is imported and ATP is exported
Role of *c*-ring	*c*-ring together with *γ*-subunit and *ε*-subunit constitutes a rotor of electromotor	*c*-ring is non-selective ion channel
Role of *b*_2_-subunit	*b*_2_-Subunit is a part of stator together with *a*-subunit	*b*_2_-Subunits represent cords that are in a twisted state and, which twists more during binding of phosphate ions; the membrane potential allows the hexamer to be dragged to the membrane
Role of *ε*-subunit	*ε*-Subunit regulates ATPase activity	*ε*-Subunit is a mechanical «stopper» of the proton channel in contracted conformation. It protonates *β*-subunit and also regulates ATPase activity in extended conformation
Role of *γ*-subunit	*γ*-Subunit represents an axis of the rotor—*c*-ring and rotates clockwise direction during ATP hydrolysis and counterclockwise direction in ATP synthesis	γ-Subunit is the rotor. It rotates counterclockwise direction 360° during energization, and then it rotates clockwise direction 360° back during the synthesis of ATP. Three Pi together with three protons are delivered to active centers at *C*-terminal part of *γ*-subunit
Role of *α*_3_ *β*_3_-hexamer	*α*_3_ *β*_3_-Hexamer has three catalytic and three non-catalytic centers; it represents a part of stator of electromotor	*α*_3_ *β*_3_-Hexamer has three catalytic and three non-catalytic centers; it is moved (dragged by *b*_2_-subunits) to the membrane during the energization process and is moved from the membrane during the deenergization process
Regulation of electron transport and proton transfer	Electron transport and proton transfer are regulated by pH changes	Electron transport through cytochrome *bc*_1_ and proton passage through proton channel in ATP synthase are regulated by pH changes and mechanically
Evidence for the model	Currently there is no working model	The direct proof of this model is the acting nanomotor of carbon nanotube which operates on the same principles

## Proposed examination and test of the mechano-chemiosmotic model

We are aware that the view presented here is not accepted by other model, but is presented here for further examination and testing, so that we may reach a final conclusion on this important problem. In order to prove the mechano-chemiosmotic model of coupling, it is necessary to perform further kinetic experiments (it is desirable to simultaneously obtain different parameters) on individual organelles in millisecond time scale:

- Cyclic volume changes (shrinkage–swelling) of intracristal space of mitochondria and lumen of chloroplast thylakoids;
- Cyclic volume changes (shrinkage–swelling) of matrix of mitochondria and stroma of chloroplasts;
- pH changes of intracristal space of mitochondria and lumen of chloroplast thylakoids;
- pH changes of matrix of mitochondria and stroma of chloroplasts (mitochondrial matrix pH of individual mitochondria undergoes spontaneous alkalization transients (Poburko et al. 2011));
- pH changes of the outer medium of mitochondria and chloroplasts—cytosol (there are pH changes data of mitochondria outer medium in *min* time scale (Kasumov et al. 1991a, b));
- Dependence of the oxidation–reduction of Cyt *c*_1_ or Cyt *f* on the swelling–shrinkage of the mitochondrial intracristal space or lumen of thylakoids (for example, this dependence may be investigated on mitoplasts);
- Inter-monomer electron transfer from the [2Fe–2S] cluster to heme *f* between the opposite dimers during shrinkage;
- Polarization–depolarization of mitochondria and thylakoids membrane potentials;
- Movement of potassium and calcium ions between the matrix and the intermembrane space of mitochondria, and between the stroma and the lumen of chloroplasts;
- Movement of potassium, calcium, and other ions through F_0_ of ATP synthase in different directions;
- Conformational changes of ATP synthase subunits during shrinkage–swelling of organelles;
- Direction of the rotation of gamma subunit of ATP synthase in energization and deenergization of mitochondria and thylakoids; and
- Rotation of *c*-ring by protons in the presence pH gradient in different sides of the membrane without ATP and other phosphate compounds.

We believe that in the presence of oxidative substrate, phosphate ions, ADP, and other essential ions for the formation of ATP, in energization of mitochondria, we will observe the following processes in the sequence:

*Polarization of inner membrane* → movement of potassium ions to the matrix, and calcium ions to the intermembrane space → swelling of the mitochondrial matrix → shrinkage of intracristal space → reduction of Cyt *c*_1_→ *depolarization of inner membrane* → movement of calcium ions into the matrix, and potassium ions to the intermembrane space → contraction of the mitochondrial matrix → ATP synthesis → swelling of intracristal space → oxidation of Cyt *c*_1_ → release of protons into the external medium (cytosol) → a repeat of cycle …

We note that Lee and Yoon (2014) have found that electron transport activity is necessary for the morphological contraction of mitochondria, and this transient morphological contraction of the mitochondrial matrix is accompanied by a reversible loss or decrease of inner membrane potential (depolarization). Kirchhoff et al. (2011) found that the width of the thylakoid lumen expands in light. Then, Kirchhoff (2013) has postulated that a dynamic light-dependent osmotic swelling of the lumen is caused by an increase in Cl^−^ concentration in this narrow space controlled by voltage-gated Cl^−^ thylakoid membrane channels.

We wait for further new experiments before reaching a final conclusion on the mechanism of ATP synthesis that is one of the most important problems in Biochemistry and Biophysics of all living cells.

## Appendix

The diameter of *c*-ring may be calculated using the following equations:

$$V = \, w \, n \, S,$$

where *V* is volume of the fluid of matrix, stroma, or cytosol, as pumped out in a minimum time period—of about 1 ms; *w* is the flow rate; *d* is the inner diameter of the *c*-ring in the its middle; *n* is the amount of ATP synthase in one mitochondrion, or a chloroplast or a bacterial cell; *S* is the cross-section of *c*-ring ($S \, = \, 3.14 \, d^{2} /4$). Thus,

$$V \, = \, w \, n \, S \, = \, w \, n \, 3.14 \, d^{2} /4,$$

where $d^{2} = \, 4 V/3.14 \, w \, n$; and then $d \, = \, \sqrt {\left( {4 V/3.14 \, w \, n} \right)} .$

Thus, inner diameter of *c*-ring (*d*) is reversibly proportional to the amount of ATP synthases (*n*) and directly proportional to the volume (*V*) of matrix, stroma, or cytosol.

## Abbreviations

ETC: Electron transport chain
pmf: Proton-motive force
ΔμH^+^: Electrochemical potential
mPTP: Mitochondrial permeability transition pore
NADP^+^: Nicotinamide adenine dinucleotide phosphate
Deh: Dehydrogenase
Cyt: Cytochrome
CoQ: Ubiquinone
Fp: Flavoprotein
PQ: Plastoquinone
PC: Plastocyanin
PS: Photosystem
Fd: Ferredoxin
FNR: Ferredoxin-NADP reductase
